# Beyond cellular detoxification: a plethora of physiological roles for MDR transporter homologs in plants

**DOI:** 10.3389/fphys.2014.00201

**Published:** 2014-05-30

**Authors:** Estelle Remy, Paula Duque

**Affiliations:** Instituto Gulbenkian de CiênciaOeiras, Portugal

**Keywords:** ATP-Binding Cassette (ABC), ion homeostasis, Major Facilitator Superfamily (MFS), membrane transporter, Multidrug And Toxin compound Extrusion (MATE), plant systems, polar auxin transport (PAT), xenobiotic detoxification

## Abstract

Higher plants possess a multitude of Multiple Drug Resistance (MDR) transporter homologs that group into three distinct and ubiquitous families—the ATP-Binding Cassette (ABC) superfamily, the Major Facilitator Superfamily (MFS), and the Multidrug And Toxic compound Extrusion (MATE) family. As in other organisms, such as fungi, mammals, and bacteria, MDR transporters make a primary contribution to cellular detoxification processes in plants, mainly through the extrusion of toxic compounds from the cell or their sequestration in the central vacuole. This review aims at summarizing the currently available information on the *in vivo* roles of MDR transporters in plant systems. Taken together, these data clearly indicate that the biological functions of ABC, MFS, and MATE carriers are not restricted to xenobiotic and metal detoxification. Importantly, the activity of plant MDR transporters also mediates biotic stress resistance and is instrumental in numerous physiological processes essential for optimal plant growth and development, including the regulation of ion homeostasis and polar transport of the phytohormone auxin.

## Introduction

Multiple Drug Resistance (MDR), the simultaneous acquisition of resistance to multiple structurally and functionally unrelated cytotoxic compounds, is a widespread biological phenomenon. One way by which a living cell can achieve MDR is by actively extruding toxic compounds. Of the five transporter families hitherto described to include multidrug efflux pumps—Small Multidrug Resistance (SMR), Resistance/Nodulation/Division (RND), ATP-Binding Cassette (ABC), Major Facilitator Superfamily (MFS), and Multidrug And Toxic compound Extrusion (MATE) (Paulsen, 2003)—only the ubiquitous ABC, MFS, and MATE are found in higher eukaryotes. As with many other conserved gene families, those of the ABC, MFS, and MATE appear significantly more expanded in plants than in bacteria, yeast or animals, with the *Arabidopsis thaliana* genome encoding around 130 ABC, 120 MFS, and 58 MATE transporters (Huala et al., 2001; Ren et al., 2004). Strikingly, the majority of the plant transporters belonging to these three families remain to be functionally characterized. We review here the available functional data on these plant transporters (summarized in Table 1), substantiating not only a role in cellular detoxification but also in a wide range of physiological processes.

**Table 1:** **Representative examples of plant MDR transporter homologs and their physiological functions**.

**Transporter**	**Species**	**Biological function(s)**	**References**
**MDR/PGP—FULL-SIZE MEMBERS OF THE ABCB SUBFAMILY OF ABC TRANSPORTERS**			
ABCB1/PGP1	*Arabidopsis thaliana*	Resistance to multiple xenobiotics including herbicides Polar auxin transport	Thomas et al., 2000; Noh et al., 2001, 2003; Windsor et al., 2003; Lin and Wang, 2005; Lewis et al., 2007
ABCB4/PGP4	*Arabidopsis thaliana*	Polar auxin transport	Santelia et al., 2005; Terasaka et al., 2005; Cho et al., 2007; Lewis et al., 2007; Wu et al., 2007; Yang and Murphy, 2009
ABCB14/PGP14	*Arabidopsis thaliana*	Polar auxin transport Stomatal closure regulation	Lee et al., 2008; Kaneda et al., 2011
ABCB15/PGP15	*Arabidopsis thaliana*	Polar auxin transport	Kaneda et al., 2011
ABCB19/PGP19	*Arabidopsis thaliana*	Polar auxin transport	Noh et al., 2001, 2003; Lin and Wang, 2005; Lewis et al., 2007
ABCB21/PGP21	*Arabidopsis thaliana*	Polar auxin transport	Kamimoto et al., 2012
**ABCC (MRP) SUBFAMILY OF ABC TRANSPORTERS**			
ABCC1/MRP1	*Arabidopsis thaliana*	Vacuolar sequestration of xenobiotic conjugates including herbicides, of excess folates and of a conjugate of ABA Tolerance to arsenic, cadmium and mercury	Lu et al., 1997; Raichaudhuri et al., 2009; Song et al., 2010; Park et al., 2012; Burla et al., 2013
ABCC2/MRP2	*Arabidopsis thaliana*	Vacuolar sequestration of xenobiotic conjugates including herbicides and of a conjugate of ABA Vacuolar sequestration of chlorophyll catabolites Tolerance to arsenic, cadmium and mercury	Lu et al., 1998; Song et al., 2010; Park et al., 2012; Burla et al., 2013
ABCC3/MRP3	*Arabidopsis thaliana*	Vacuolar sequestration of xenobiotic conjugates including herbicides Vacuolar sequestration of chlorophyll catabolites	Tommasini et al., 1998
MRP4/ABCC4	*Arabidopsis thaliana*	Vacuolar sequestration of excess folates Regulation of stomatal movements	Klein et al., 2004
ABCC5/MRP5	*Arabidopsis thaliana*	Regulation of seed phytate content Regulation of stomatal movements	Gaedeke et al., 2001; Klein et al., 2003; Suh et al., 2007; Nagy et al., 2009; Kang et al., 2011
MRP3	*Zea mays*	Vacuolar anthocyanin accumulation	Goodman et al., 2004
MRP4	*Zea mays*	Regulation of seed phytate content	Shi et al., 2007
**PDR—FULL-SIZE MEMBERS OF THE ABCG SUBFAMILY OF ABC TRANSPORTERS**			
ABCG30/PDR2	*Arabidopsis thaliana*	Root exudation of phytochemicals	Badri et al., 2009
ABCG36/PDR8	*Arabidopsis thaliana*	Resistance to cadmium and sodium Resistance to fungal and bacterial pathogens Resistance to synthetic auxins IBA transport Root exudation of phytochemicals	Kobae et al., 2006; Stein et al., 2006; Kim et al., 2007, 2010; Strader and Bartel, 2009; Badri et al., 2012; Underwood and Somerville, 2013; Xin et al., 2013
ABCG37/PDR9	*Arabidopsis thaliana*	Resistance to synthetic auxins IBA transport	Ito and Gray, 2006; Ruzicka et al., 2010; Badri et al., 2012
ABCG40/PDR12	*Arabidopsis thaliana*	Sclareol resistance ABA transport Lead detoxification	Campbell et al., 2003; Lee et al., 2005; Kang et al., 2010
PDR1	*Nicotiana plumbaginifolia*	Terpene transport Basal defense	Jasinski et al., 2001; Stukkens et al., 2005
PDR5	*Nicotiana tabacum*	Herbivore defense	Bienert et al., 2012
TUR2	*Spirodella polyrhiza*	Terpene transport including sclareol	van den Brule et al., 2002
LR34	*Triticum* spp.	Resistance to fungal pathogens	Krattinger et al., 2009; Risk et al., 2013
**MAJOR FACILITATOR SUPERFAMILY (MFS)**			
NRT1.1/CHL1	*Arabidopsis thaliana*	Nitrate sensing Regulation of nascent organ development, stomatal opening, seed germination and root architecture	Guo et al., 2001, 2003; Alboresi et al., 2005; Remans et al., 2006; Walch-Liu and Forde, 2008; Ho et al., 2009
Pht1;1	*Arabidopsis thaliana*	Pi uptake under Pi-sufficient and Pi-deficient environmental conditions	Shin et al., 2004
Pht1;4	*Arabidopsis thaliana*	Pi uptake under Pi-sufficient and Pi-deficient environmental conditions	Misson et al., 2004; Shin et al., 2004
Pht1;5	*Arabidopsis thaliana*	Pi mobilization from phosphorous source to sink organs	Nagarajan et al., 2011
Pht1;8	*Arabidopsis thaliana*	Pi uptake under phosphate starvation	Remy et al., 2012
Pht1;9	*Arabidopsis thaliana*	Pi uptake under phosphate starvation	Remy et al., 2012
Pht4;1	*Arabidopsis thaliana*	Basal defense against pathogens	Wang et al., 2011
Pht4;2	*Arabidopsis thaliana*	Root starch accumulation Leaf size	Irigoyen et al., 2011
Pht4;6	*Arabidopsis thaliana*	Tolerance to salt stress Biotic stress resistance	Cubero et al., 2009; Hassler et al., 2012
STP1	*Arabidopsis thaliana*	Uptake of hexoses by seeds and seedlings Monosaccharide import into guard cells	Sherson et al., 2000
ZIF1	*Arabidopsis thaliana*	Zinc tolerance via vacuolar sequestration of nicotianamine Iron homeostasis	Haydon and Cobbett, 2007; Haydon et al., 2012
ZIF2	*Arabidopsis thaliana*	Zinc tolerance via vacuolar sequestration	Remy et al., 2014
ZIFL1	*Arabidopsis thaliana*	Resistance to 2,4-D Cesium sensitivity Modulation of polar auxin transport Regulation of stomatal apertures and drought stress tolerance	Remy et al., 2013a,b
**MATE FAMILY**			
ADP1	*Arabidopsis thaliana*	Regulation of local auxin biosynthesis and plant architecture	Li et al., 2014
ADS1	*Arabidopsis thaliana*	Negative regulator of plant biotic stress resistance	Sun et al., 2011
ALF5	*Arabidopsis thaliana*	Root protection from inhibitory compounds	Diener et al., 2001
DTX15/FFT	*Arabidopsis thaliana*	Flavonoid transport Root growth, seed development and germination, and pollen development	Thompson et al., 2010
EDS5	*Arabidopsis thaliana*	SA-dependent signaling for plant disease resistance	Nawrath et al., 2002; Serrano et al., 2013; Yamasaki et al., 2013
FRD3	*Arabidopsis thaliana*	Citrate-mediated iron shoot/root translocation Zinc tolerance	Durrett et al., 2007; Pineau et al., 2012
MATE	*Arabidopsis thaliana*	Citrate-mediated aluminum tolerance	Liu et al., 2009
TT12	*Arabidopsis thaliana*	Vacuolar transport of proanthocyanidin precursors in seed-coat cells	Debeaujon et al., 2001; Marinova et al., 2007
ZRZ	*Arabidopsis thaliana*	Organ initiation	Burko et al., 2011
MATE1	*Eucalyptus camaldulensis*	Citrate-mediated aluminum tolerance	Sawaki et al., 2013
AACT1	*Hordeum vulgare*	Citrate-mediated aluminum tolerance	Furukawa et al., 2007; Zhou et al., 2013
MATE1	*Lotus japonicum*	Citrate-mediated iron translocation to nodule tissues	Takanashi et al., 2013
MATE1	*Medicago truncatula*	Vacuolar transport of proanthocyanidin precursors	Zhao and Dixon, 2009
MATE2	*Medicago truncatula*	Vacuolar transport of anthocyanins	Zhao et al., 2011
JAT1	*Nicotiana tabacum*	Vacuolar sequestration of nicotine	Morita et al., 2009
MATE1	*Nicotiana tabacum*	Vacuolar sequestration of nicotine	Shoji et al., 2009
MATE2	*Nicotiana tabacum*	Vacuolar sequestration of nicotine	Shoji et al., 2009
FRDL4	*Oryza satviva*	Citrate-mediated aluminum tolerance	Yokosho et al., 2011
FRDL1	*Oryza satviva*	Citrate-mediated iron shoot/root translocation	Yokosho et al., 2009
MATE1	*Oryza satviva*	Negative regulator of biotic stress and arsenic resistance Plant development	Tiwari et al., 2014
MATE2	*Oryza satviva*	Negative regulator of biotic stress and arsenic resistance Plant development	Tiwari et al., 2014
Alt(SB)	*Sorghum bicolor*	Citrate-mediated aluminum tolerance	Magalhaes et al., 2007
MATE	*Vigna umbellata*	Citrate-mediated aluminum tolerance	Yang et al., 2011

## ABC transporters

ABC transporters hydrolyze ATP to transport substrate molecules across cellular membranes. All membrane-bound ABC proteins consist of a double set of two basic structural modules: a transmembrane domain (TMD), typically containing six membrane-spanning segments, and a cytoplasmic nucleotide-binding domain (NBD), containing the ABC. The so-called full-size ABC transporters contain all four elements in a single polypeptide chain, while half-size transporters combine two TMD-NBD units as homo- or heterodimers (Higgins et al., 1986). In plants, full-size ABC transporters have been better studied. Of the 53 *Arabidopsis* full-size members, all but two can be divided into three groups: the multidrug resistance (MDR) or P-glycoproteins (PGP) belonging to the ABCB subfamily, the multidrug resistance-associated protein (MRP)/ABCC subfamily, and the pleiotropic drug resistance (PDR) of the ABCG subfamily (Sanchez-Fernandez et al., 2001).

ABC transporters came into spotlight when the MDR1 PGP was found to determine MDR of cancer cells (Chen et al., 1986), and in fact early studies of plant ABCs focused on a potential role in cell detoxification. The sole MDR-like transport mechanism reported in plants so far arose from the functional characterization of one of the 21 full-size members of the ABCB family, the *Arabidopsis AtABCB1* gene, whose cloning disclosed the occurrence of ABCs in plants (Dudler and Hertig, 1992). Indeed, ectopic expression of *AtABCB1* in *Arabidopsis* conferred enhanced resistance to multiple xenobiotics, namely to various classes of herbicides including dicamba, pendimethalin, oryzalin, or monosodium acid methanearsonate, pointing to a resistance mechanism relying on decreased retention or increased active xenobiotic efflux from cells (Thomas et al., 2000; Windsor et al., 2003). Several detailed studies also demonstrated that AtABCB1 and the closely related AtABCB19 are required for polar transport of auxin, the major growth phytohormone, by facilitating cellular efflux of indole-3-acetic acid (IAA), its predominant endogenous form (Noh et al., 2001, 2003; Lin and Wang, 2005; Lewis et al., 2007). Another extensively studied ABCB transporter, AtABCB4, was implicated in root shootward auxin transport and appears to function in both cellular IAA efflux and influx (Santelia et al., 2005; Terasaka et al., 2005; Cho et al., 2007; Lewis et al., 2007; Wu et al., 2007; Yang and Murphy, 2009). Interestingly, recent findings indicate that AtABCB4 is also able to mediate cellular influx of 2,4-dichlorophenoxyacetic acid (2,4-D), rendering the carrier a target of the herbicidal activity of this synthetic auxin (Kubes et al., 2012). An *Arabidopsis* AtABCB4 homolog, AtABCB21, has also been described to mediate IAA import/export, depending on the cytoplasmic concentration of the phytohormone (Kamimoto et al., 2012), while AtABCB14 and AtABCB15 have been associated with polar auxin transport (PAT) in inflorescence stems (Kaneda et al., 2011). Thus, all plant full-size ABCB (MRP/PGP) transporters characterized to date contribute to PAT in vegetative tissues, directing long-distance auxin transport in mature plants. Nevertheless, AtABCB14 was first reported as a malate importer regulating stomatal closure (Lee et al., 2008), suggesting that full-size ABCB carriers may play important roles in other key processes.

ABCC (MRP) transporters, which have been typically associated with detoxification processes, were first identified in human drug-resistant cancer cells (Cole et al., 1992). Most of the 15 *Arabidopsis* members are localized at the vacuolar membrane (Rea, 2007), representing the only tonoplastic full-size ABC transporters described to date (Kang et al., 2011). A common cellular detoxification strategy in plants is to sequester toxic compounds in the vacuole to avoid deleterious effects on cytosolic metabolism. Early studies showed that plant vacuolar accumulation of glutathionated xenobiotics is ATP-dependent (Martinoia et al., 1993), prompting the identification of the first plant ABCC transporters. Indeed, AtABCC1, AtABCC2, and AtABCC3 are implicated in vacuolar sequestration of conjugated xenobiotics such as herbicides, with the latter two transporters functioning also in endogenous chlorophyll catabolite detoxification (Lu et al., 1997, 1998; Tommasini et al., 1998). Importantly, AtABCC1 and AtABCC2 were recently described as phytochelatin transporters with overlapping functions in plant tolerance to the metalloid arsenic and the heavy metals cadmium and mercury (Song et al., 2010; Park et al., 2012). There is also evidence pointing to a role of AtABCC3 and AtABCC6 in responses to cadmium stress (Tommasini et al., 1998; Gaillard et al., 2008). Furthermore, both AtABCC1 (Raichaudhuri et al., 2009) and AtABCC4 (Klein et al., 2004) are involved in vacuolar sequestration of excess folates, whereas AtABCC5 is a high-affinity inositol hexakisphosphate (InsP_6_) transporter modulating seed phytate content (Nagy et al., 2009) as its maize homolog ZmMRP4 (Shi et al., 2007). Interestingly, both AtABCC4 (Klein et al., 2004) and AtABCC5 (Gaedeke et al., 2001; Klein et al., 2003; Suh et al., 2007) regulate stomatal movements, which in the case of AtABCC5 could be linked to its InsP_6_ transport activity (Kang et al., 2011). Finally, ZmMRP3 was shown to affect vacuolar anthocyanin accumulation (Goodman et al., 2004), and a recent study implicated AtABCC1 and AtABCC2 in vacuolar sequestration of a conjugate of the phytohormone abscisic acid (ABA) (Burla et al., 2013). It is therefore clear that plant ABCC transporters are involved in a range of processes beyond detoxification, such as the transport of primary and storage compounds or hormones and the control of stomatal apertures.

PDR proteins are specific to plants and fungi and in *Arabidopsis* comprise the 15 full-size members of the ABCG subfamily, which uniquely among ABCs feature a reverse organization of the NBD and TMD domains in each unit (Crouzet et al., 2006). All PDRs characterized so far are plasma membrane transporters (Kang et al., 2011), and the first to be identified in plants, the *Spirodella polyrhiza* SpTUR2 and the *Nicotiana plumbaginifolia* NpPDR1, mediate the transport of terpenes (Jasinski et al., 2001; van den Brule et al., 2002). SpTUR2 expression in *Arabidopsis* confers resistance to the diterpenoid sclareol, as does the *Arabidopsis* AtABCG40 (Campbell et al., 2003) later shown to function in cellular uptake of the sesquiterpenoid ABA (Kang et al., 2010). Intriguingly, AtABCG40 also mediates detoxification of the heavy metal lead via a glutathione-independent process (Lee et al., 2005). Another PDR transporter, AtABCG36, is involved in cadmium (Kim et al., 2007) and sodium toxicity (Kim et al., 2010) resistance. Following the findings that NpPDR1 secretes antifungal terpenoids (Jasinski et al., 2001) and contributes to basal plant defense (Stukkens et al., 2005), AtABCG36 was identified as a key factor in the resistance to fungal and bacterial pathogens (Kobae et al., 2006; Stein et al., 2006; Underwood and Somerville, 2013; Xin et al., 2013). Moreover, both AtABCG36 and AtABCG37 excrete a range of synthetic auxins, including 2,4-D, and indole-3-butyric acid (IBA), the natural IAA precursor (Ito and Gray, 2006; Strader and Bartel, 2009; Ruzicka et al., 2010). Interestingly, AtABCG36, AtABCG37, and AtABCG30 are involved in root exudation of phytochemicals (Badri et al., 2009, 2012). Recent studies implicate the tobacco NtPDR5 in herbivore defense (Bienert et al., 2012), while the wheat PDR carrier LR34 confers resistance to fungal pathogens (Krattinger et al., 2009; Risk et al., 2013). Thus, plant full-size ABCG proteins play a preponderant role in metal and xenobiotic detoxification as well as in biotic stress resistance, but also fulfill functions in phytohormone transport.

## MFS transporters

After ABCs, the MFS represents the second largest group of transporters on earth. All MFS proteins are single-polypeptide secondary carriers capable of transporting only small molecules across membranes via a uniport, symport, or antiport mechanism using chemiosmotic gradients as energy source (Pao et al., 1998). Their protein domain organization typically consists of two TMDs, each composed of six membrane-spanning segments, flanking a central hydrophilic pore (Goswitz and Brooker, 1995).

The few plant MFS members characterized to date have been essentially implicated in sugar (Buttner, 2007), or nitrate and oligopeptide (Tsay et al., 2007) transport. The first monosaccharide transporter identified in higher plants was the *Arabidopsis* STP1 (Sauer et al., 1990), which is able to transport a wide range of hexoses via a proton symport mechanism (Boorer et al., 1994) and has reported functions in sugar uptake by seeds and seedlings (Sherson et al., 2000) as well as by guard cells (Stadler et al., 2003). A few other *Arabidopsis* MFS sugar transporters have been functionally characterized, such as PLT5, a broad-spectrum H^+^-symporter for polyols as well as for different hexoses and pentoses in sink tissues (Klepek et al., 2005; Reinders et al., 2005). As for plant MFS nitrate transporters, by far the best characterized is AtNRT1.1 (CHL1) that functions as a nitrate sensor (Ho et al., 2009). NRT1.1 possesses dual-affinity nitrate uptake activity (Liu et al., 1999) and has been assigned a variety of signaling functions, including in the modulation of nascent organ development (Guo et al., 2001), stomatal opening (Guo et al., 2003), seed germination (Alboresi et al., 2005), and root architecture (Remans et al., 2006; Walch-Liu and Forde, 2008). Importantly, this carrier also represses lateral root growth at low nitrate availability by promoting shootward auxin transport out of these roots, thus connecting nutrient sensing and auxin-dependent developmental adaptation (Krouk et al., 2010).

Furthermore, plant MFS transporters belonging to the Pht1 and Pht4 families mediate high- and low-affinity inorganic phosphate (Pi) transport, respectively (Guo et al., 2008; Nussaume et al., 2011). Of the nine *Arabidopsis* Pht1 transporters, those characterized so far are plasma-membrane-localized, with Pht1;1, Pht1;4, Pht1;8, and Pht1;9 ensuring environmental Pi acquisition (Misson et al., 2004; Shin et al., 2004; Remy et al., 2012), while Pht1;5 mobilizes Pi from phosphorous source to sink organs (Nagarajan et al., 2011). On the other hand, the six *Arabidopsis* Pht4 members are suggested to mediate Pi transfer across internal cellular membranes (Guo et al., 2008), with the plastidic Pht4;1 and Pht4;2 influencing basal defense against pathogens (Wang et al., 2011) and starch accumulation and leaf size (Irigoyen et al., 2011), respectively. Finally, the Golgi-localized Pht4;6 determines salt tolerance and biotic stress resistance, affecting also plant growth and development (Cubero et al., 2009; Hassler et al., 2012).

A role for the MFS in plant metal homeostasis is also beginning to emerge. The *Arabidopsis* tonoplast-localized ZIF1, initially described as a transporter involved in basal tolerance to the heavy metal zinc (Haydon and Cobbett, 2007), was later additionally implicated in iron homeostasis and its substrate identified as the low molecular mass metal chelator, nicotianamine (Haydon et al., 2012). Very recently, the ZIF2 carrier was reported to also sustain zinc tolerance in *Arabidopsis* by mediating its root vacuolar sequestration. Interestingly, high zinc favors an intron retention event in the *ZIF2* 5′UTR, promoting translation of the mRNA to enhance plant tolerance to the metal (Remy et al., 2014). By contrast, a close *Arabidopsis* ZIF1 homolog, ZIFL1, does not function in zinc homeostasis but instead confers resistance to 2,4-D and sensitivity to the heavy metal cesium. This transporter exhibits H^+^-coupled K^+^ transport activity and fulfills two distinct biological functions—while the full-length ZIFL1 protein is a root tonoplastic transporter modulating shootward auxin transport, a truncated splice form is targeted to the plasma membrane of guard cells and regulates drought stress tolerance (Remy et al., 2013b). The functional characterization of the ZIF2 and ZIFL1 transporters has hence revealed striking examples of the biological impact of alternative splicing in plants, which remains largely unknown (Carvalho et al., 2013).

## MATE transporters

MATE transporters comprise the most recently identified of multidrug transporter families (Brown et al., 1999). They are characterized by the presence of 12 putative transmembrane segments and like MFSs are secondary active carriers that depend on electrochemical gradients for their activity. Plant MATEs are thought to function as H^+^-coupled antiporters and reportedly localize at the plasma membrane or the tonoplast, carrying a diverse range of compounds.

Transport activity for plant MATEs was first demonstrated for the *Arabidopsis* DTX1 and ALF5. When heterologously expressed in *Escherichia coli*, AtDTX1 serves as an efflux carrier for the antibiotic norfloxacin, ethidium bromide, the plant-derived alkaloids berberine, and palmatine as well as cadmium (Li et al., 2002). Genetic analysis of AtALF5, whose expression in yeast confers resistance to tetramethylammonium, revealed a role in root protection from inhibitory compounds (Diener et al., 2001). However, the first plant MATE transporter to be identified, AtTT12, was implicated in the vacuolar accumulation of flavonoids, a class of plant-specific secondary metabolites, in the seed coat (Debeaujon et al., 2001), and later confirmed to be a tonoplast-localized vacuolar flavonoid/H^+^-antiporter active in proanthocyanidin-accumulating seed-coat cells (Marinova et al., 2007). Several other studies have corroborated a role for MATEs in the vacuolar accumulation of proanthocyanins and anthocyanins in different plant tissues, including in *Arabidopsis*, *Medicago truncatula*, tomato, and grapevine (Mathews et al., 2003; Gomez et al., 2009; Zhao and Dixon, 2009; Thompson et al., 2010; Zhao et al., 2011). MATEs have also been shown to mediate vacuolar transport of the major alkaloid nicotine in tobacco cells (Morita et al., 2009; Shoji et al., 2009).

Importantly, vital roles for MATE transporters in plant tolerance to the heavy metal aluminum (Al) have been established. Plants cope with Al phytotoxic concentrations in the rhizosphere by releasing organic anions such as citrate that form stable non-toxic complexes with the metal (Magalhaes, 2010), and MATEs have been identified as major determinants of this Al tolerance strategy in sorghum (Magalhaes et al., 2007), barley (Furukawa et al., 2007), and rice (Yokosho et al., 2011). Citrate transporters of the MATE family have also been linked to Al tolerance in *Arabidopsis*, maize, wheat, rye, rice bean, or *Eucalyptus camaldulensis*, with a few of these MATEs conferring Al resistance when heterologously expressed in other plant species (Liu et al., 2009; Ryan et al., 2009; Maron et al., 2010; Yokosho et al., 2010; Yang et al., 2011; Sawaki et al., 2013; Zhou et al., 2013). Interestingly, citrate transport by AtFRD3 and OsFRDL1 is required for iron root/shoot translocation in *Arabidopsis* (Durrett et al., 2007) and rice (Yokosho et al., 2009), respectively. More recently, AtFDR3 was implicated in plant zinc homeostasis (Pineau et al., 2012), while a citrate MATE transporter from the model legume *Lotus japonica* assists in iron translocation to nodule tissues (Takanashi et al., 2013).

Plant MATEs also function in the response to pathogen infection. An early study revealed a role for AtEDS5 in salicylic acid (SA)-dependent disease resistance signaling (Nawrath et al., 2002). Subsequent findings that the transporter mediates SA export from the chloroplast, where synthesis of the signaling molecule occurs, provided mechanist insight into EDS5's control of plant disease tolerance (Serrano et al., 2013; Yamasaki et al., 2013). Another *Arabidopsis* MATE involved in SA-mediated pathogen response is ADS1, a negative regulator of plant biotic stress resistance (Sun et al., 2011). More recently, heterologous expression of two rice MATE genes in *Arabidopsis* was reported to affect not only pathogen susceptibility, but also arsenic sensitivity and plant development (Tiwari et al., 2014). Functional characterization of another two plant MATE transporters, AtZRZ and AtADP1, substantiated a role in development, namely in plant architecture and organ initiation (Burko et al., 2011; Li et al., 2014).

## Concluding remarks

Plant MDR transporter homologs substantially contribute to cellular detoxification of metals and xenobiotic compounds as well as to biotic stress resistance. Besides these rather expected functions, most of the available functional data show that these transporters also fulfill essential roles in numerous physiological processes, ranging from hormone transport to the regulation of ion homeostasis and stomatal movements, thus modulating plant growth and development. Hitherto, and to the best of our knowledge, an MDR transporter *sensu stricto*, i.e., a membrane pump that exclusively catalyzes the cellular efflux of a broad range of chemically distinct xenobiotics, has not been identified in plants.

**Figure 1:**
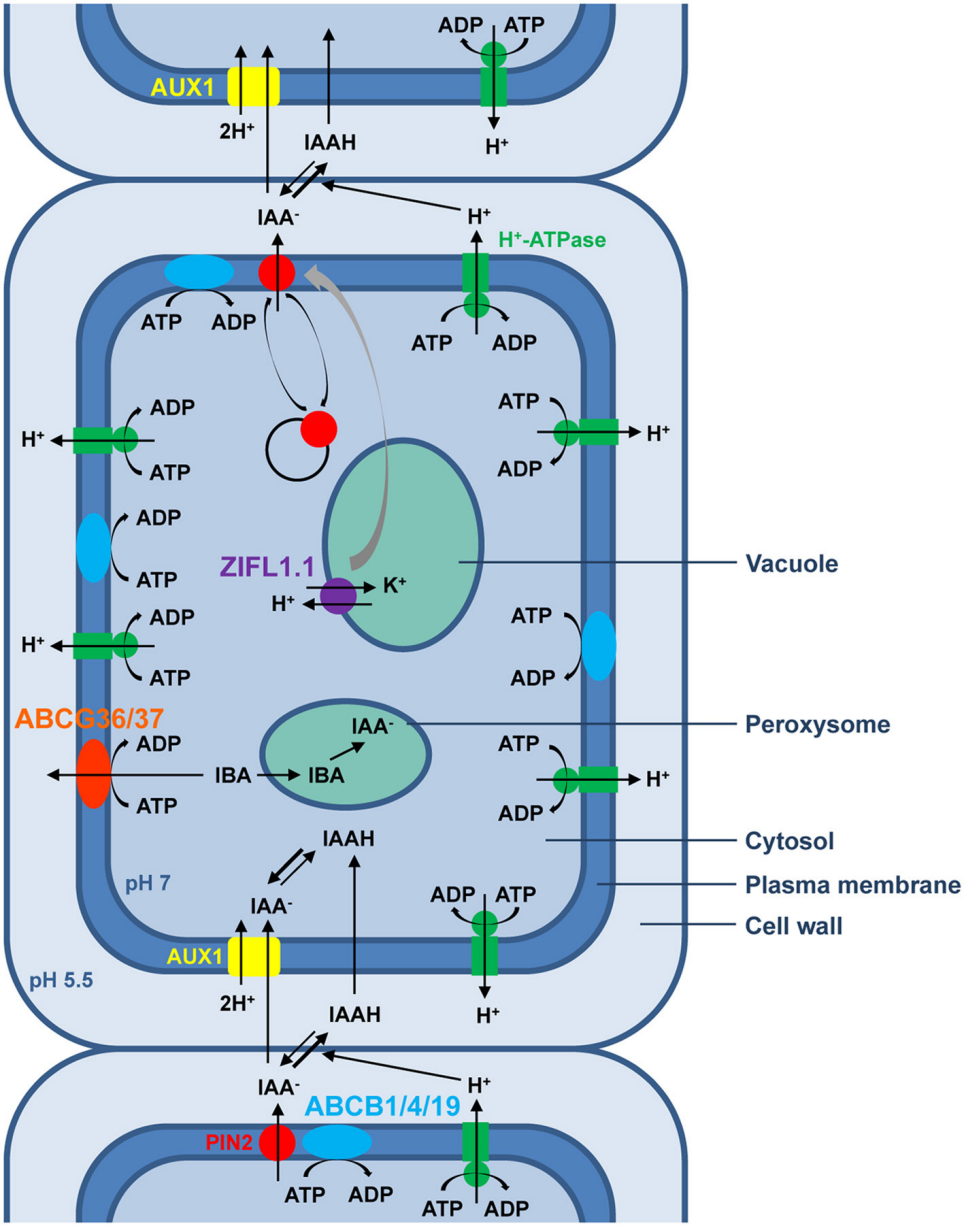
**Schematic representation of polar auxin transport (PAT) in epidermal cells of the *Arabidopsis* root tip**. According to the chemiosmotic hypothesis, the proton gradient generated primarily by plasma membrane H^+^-ATPases between the neutral cytoplasm and the acidic extracellular space drives the polarized auxin cell-to-cell movement. In the acidic apoplastic environment, a fraction of the weak acid IAA exists in its undissociated form, which can passively diffuse through the plasma membrane inside the cell. By contrast, the non-lipophilic and therefore less permeable proton-dissociated auxin fraction requires the amino acid permease-like AUX1, which catalyzes proton symport activity, to enter the cell. In the neutral cytosolic environment, IAA exists mainly in its membrane-impermeant anionic form that requires active transport to exit the cell. Hitherto, two distinct protein families whose members possess IAA-exporting activity have been associated with cellular polar auxin efflux. The best characterized auxin efflux carriers are members of the unique and plant-specific PIN protein family, believed to be secondary transporters energized by proton gradients. By contrast, some plant homologs of the human MDR/PGP transporters belonging to the ABCB subfamily, such as ABCB1, ABCB4, and ABCB19, have been implicated in ATP-energized auxin efflux. Although activity of ABCBs and the asymmetrical localization of AUX1 facilitates directionality of auxin transport, the bias, and rate of shootward auxin transport are mainly attributable to the highly regulated polar localization of the PIN2 transporter. Dynamic polar sorting of PIN2 at the plasma membrane is sustained by repeated steps of endocytic internalization and recycling back to the plasma membrane via exocytosis. In addition, potassium transport activity of the ZIFL1.1 tonoplastic carrier exerts a protective effect on PIN2 plasma-membrane stability. The hormonal activity of the auxin precursor IBA requires its conversion to IAA through β-oxydation in the peroxysome. Two members of the G-family of ABC transporters, ABCG36 and ABCG37, localize to the outward face of root epidermal cells and efflux IBA from root cells.

Besides global ion homeostasis regulation, in which all three plant MDR transporter families have long been implicated, the physiological process reported to date to require the activity of the largest number of MDR transporter homologs is PAT, as exemplified in Figure 1 for root shootward auxin transport in *Arabidopsis*. Many key aspects of plant development are regulated by PAT, whose rate-limiting step, cellular IAA efflux, relies primarily on the regulated polar localization of PIN transporters at the plasma membrane (Petrasek et al., 2006; Wisniewska et al., 2006). Furthermore, all six characterized members of the *Arabidopsis* PGP/ABCB subfamily contribute to PAT. While PINs and ABCBs define two distinct IAA efflux systems, roles for ABCBs in providing IAA to PINs for vectorial transport (Mravec et al., 2008) or in stabilizating PINs at the plasma membrane to enhance IAA specificity (Blakeslee et al., 2007; Titapiwatanakun et al., 2009) have been demonstrated. Thus, both efflux transport systems act concertedly to generate and maintain auxin gradients. Importantly, auxin distribution can also be influenced by directional IBA transport across the plasma membrane, a role fulfilled by AtABCG36 and AtABCG37 that act redundantly at outermost root plasma membranes to export IBA from cells, thereby contributing to IBA and auxinic compound sensitivity and regulating multiple aspects of primary root development (Ruzicka et al., 2010). Apart from ABC transporters, an *Arabidopsis* MFS member was recently shown to act as a general positive modulator of PAT by stabilizing PIN plasma-membrane abundance—ZIFL1.1 activity is required for fine-tuning of root shootward auxin transport rates under conditions normally triggering PIN degradation and regulates lateral root growth and root gravitropic responses (Remy et al., 2013a,b). Moreover, the MATE AtADP1 transporter appears to regulate local auxin levels in meristematic tissues to control lateral organ growth in *Arabidopsis* (Li et al., 2014). Future characterization of additional plant MFS and MATE members will likely unveil a broader role for these transporter families in PAT.

### Conflict of interest statement

The authors declare that the research was conducted in the absence of any commercial or financial relationships that could be construed as a potential conflict of interest.
